# Clinical Features and Efficacy Analysis of Redundant Nerve Roots

**DOI:** 10.3389/fsurg.2021.628928

**Published:** 2021-11-01

**Authors:** Jianzhong Xu, Yong Hu

**Affiliations:** Department of Spinal Surgery, Ningbo No.6 Hospital, Ningbo, China

**Keywords:** redundant nerve roots, lumbar vertebrae, instrumented fusion, posterior lumbar interbody fusion, magnetic resonance imaging

## Abstract

**Introduction:** Redundant nerve roots (RNRs) are common finding in lumbar spinal stenosis patients. Up to now, many relevant studies were carried out on the mechanism, pathogenic factors, and clinical features of redundant nerve roots. However, there are few studies on the surgical methods. In this study, posterior lumbar interbody fusion and internal fixations were used in 30 patients with RNRs in our hospital. Moreover, we also proposed new ideas about different types and subtypes of RNRs using patterns and their corresponding MRI images.

**Methods:** Thirty patients with lumbar spinal stenosis and RNRs were enrolled in this study and underwent surgery between January 2009 and December 2014. Redundant nerve roots are identified as elongated, tortuous, or serpiginous nerve roots present in the subarachnoid space on sagittal T2-weighted magnetic resonance imaging (MRI) studies. Patients were treated with posterior decompression, intervertebral disc resection, and instrumented interbody fusion. The age, sex, disease course, operative time, intraoperative blood loss, operative segments were recorded. Outcome measures recorded to identify symptom improvement included pre-operative and post-operative visual analog scale (VAS), pre-operative and post-operative Oswestry Disability Index (ODI) and pre-operative and post-operative Japanese Orthopedic Association (JOA) scores.

**Results:** VAS back pain, VAS leg pain VAS, ODI, and JOA with standard deviations were 6.4 ± 0.9, 7.1 ± 0.8, 43.0 ± 2.2, and 10.3 ± 2.6, respectively. At 3 months post-operatively, VAS back pain, VAS leg pain VAS, ODI, and JOA with standard deviations were 1.4 ± 0.5, 1.6 ± 0.6, 13.0 ± 1.6, and 25.0 ± 1.8, respectively. Nerve redundancy resolved in all cases on post-operative MRI.

**Conclusion:** Posterior lumbar laminectomy and instrumented interbody fusion relieves low back and leg pain in patients with lumbar spinal stenosis and RNRs and can alleviate the tortuous appearance of the cauda equina in the decompressed segment.

## Introduction

**Figure 1:**
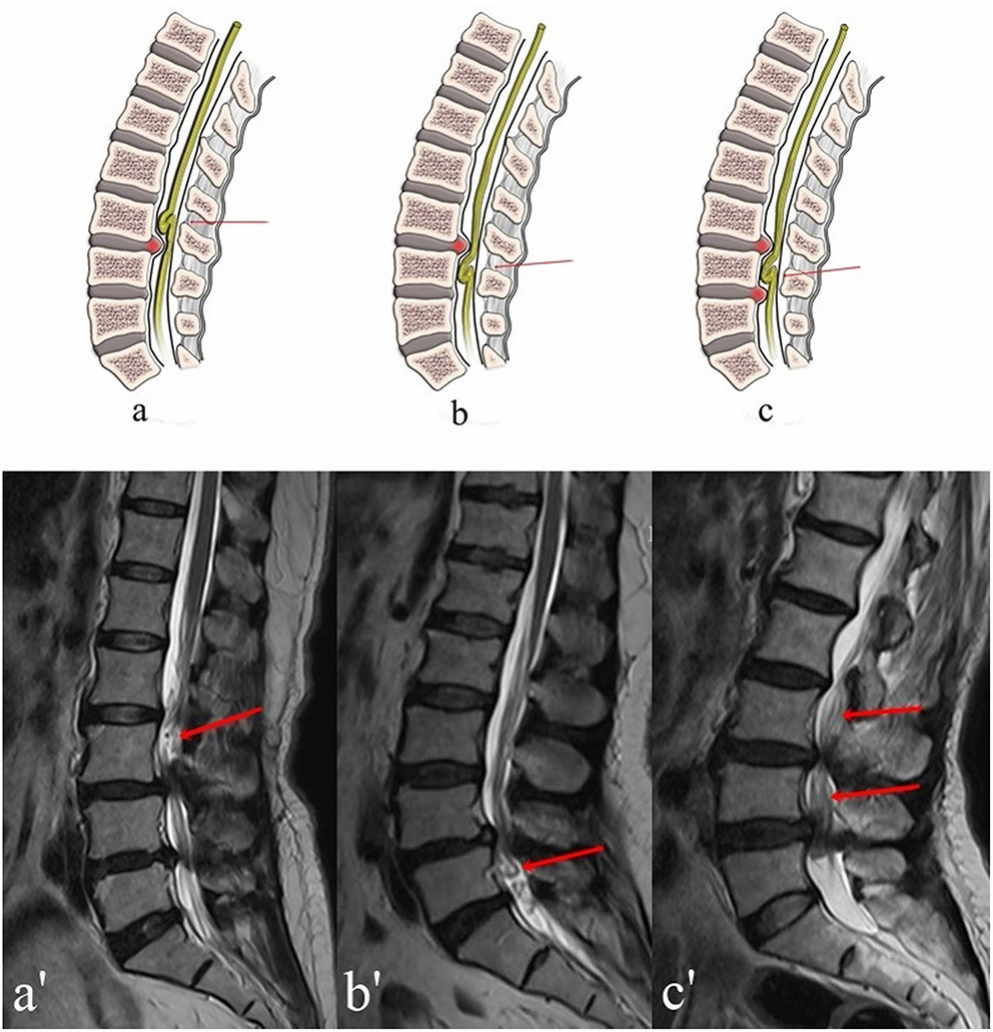
Three possible different location of RNRs with corresponding MRI images. **(A,A′)** Upper: redundant nerve roots (red arrow) above the level of maximal stenosis. **(B,B′)** Lower: redundant nerve roots (red arrow) below the level of maximal stenosis. **(C,C′)** Intermediate: redundant nerve roots (red arrows) between two levels with severe stenosis.

**Figure 2:**
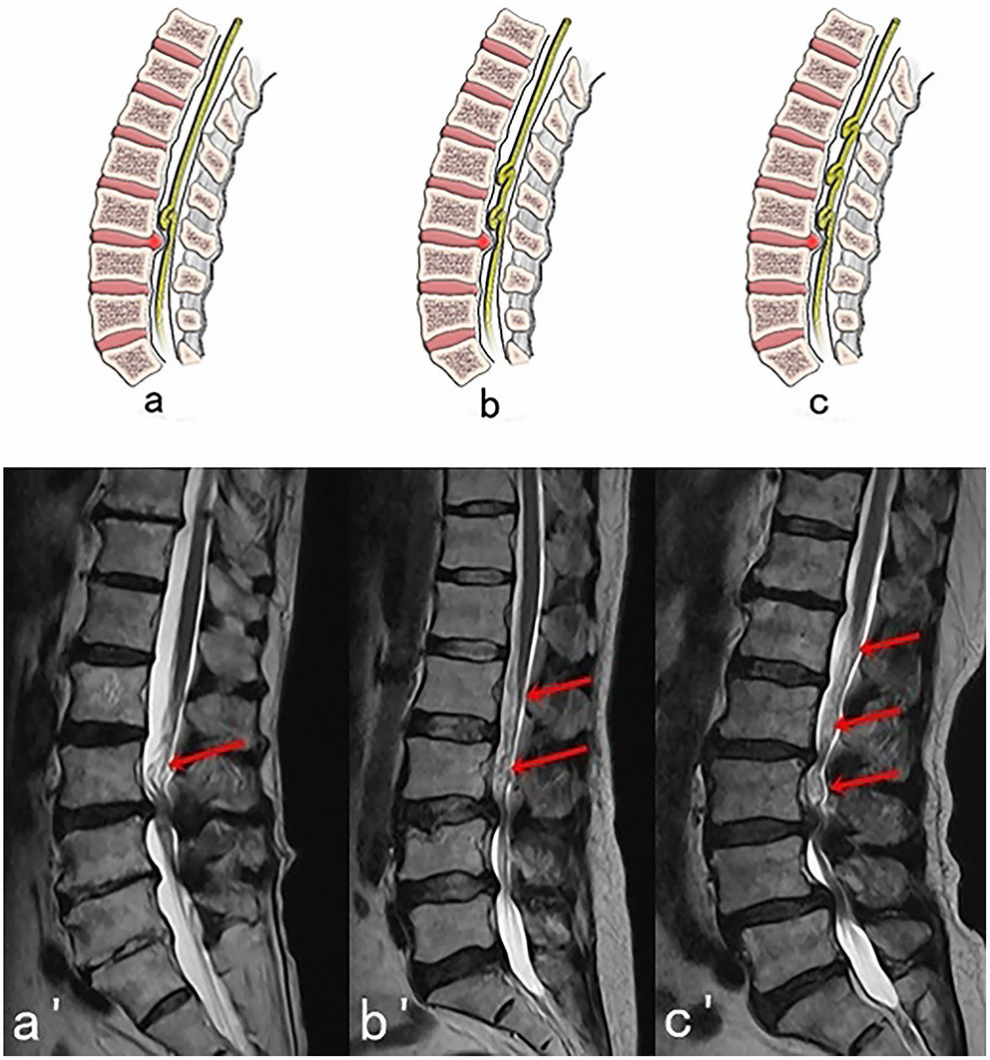
Three possible subtypes of Upper RNRs (RNRs which are above the stenosis) with corresponding MRI images. **(A,A′)** One segment of RNRs (red arrow) are above the stenosis. **(B,B′)** Two segments of RNRs (red arrows) are above the stenosis. **(C,C′)** Three segments of RNRs (red arrows) are above the stenosis.

**Figure 3:**
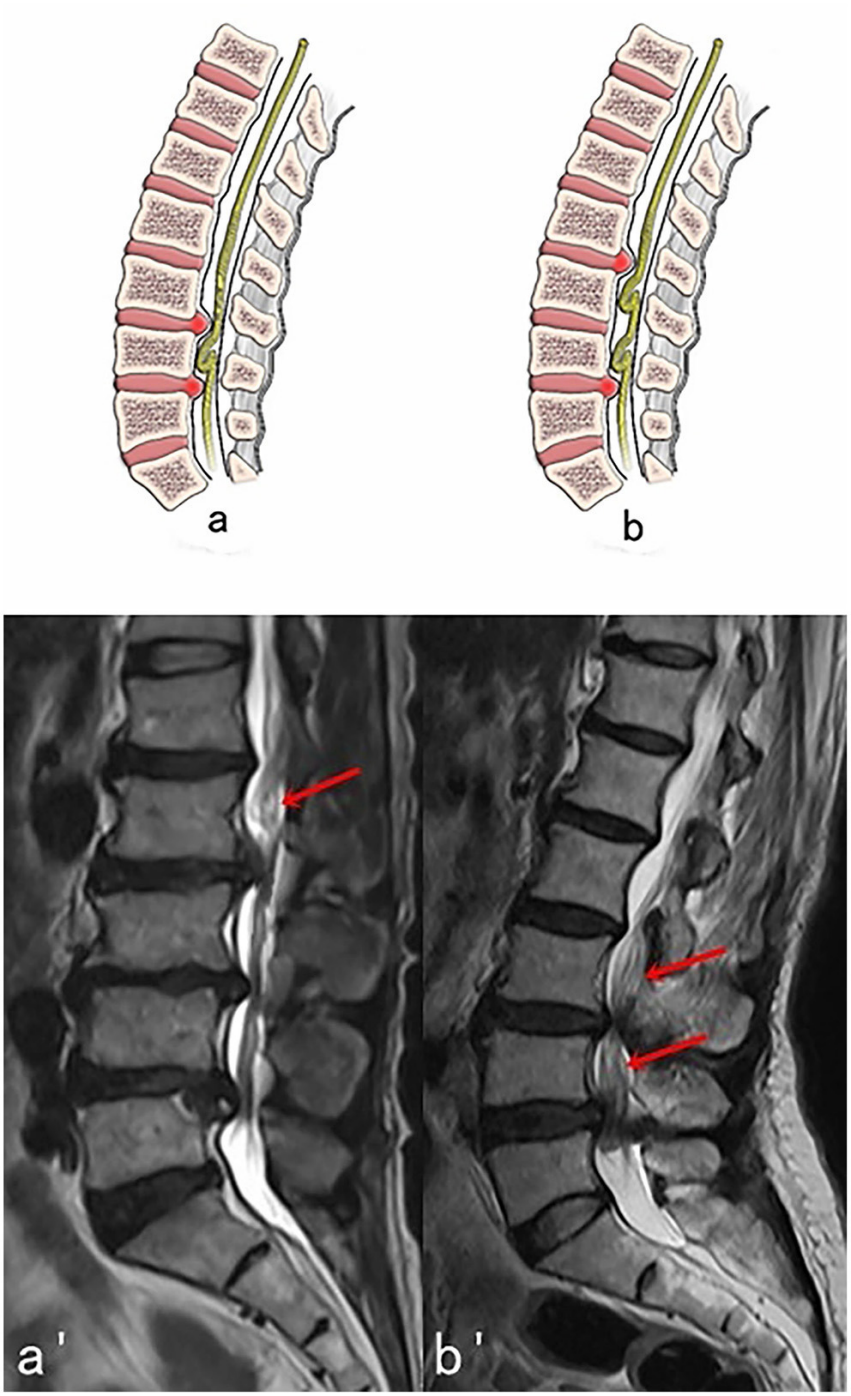
Two possible subtypes of Intermediate RNRs (RNRs which are between the stenosis) with corresponding MRI images. **(A,A′)** One segment of RNRs (red arrow) are between the stenosis. **(B,B′)** Two segments of RNRs (red arrows) are between the stenosis.

Redundant nerve roots (RNRs) were first described by Verbiest (1), and subsequently named by Cressman and Pawl (2). RNRs are characterized by a tortuosity of elongated and enlarged nerve roots in the subarachnoid space of the lumbar spine. The reported prevalence of RNRs varies, with some researchers determining prevalence values of 33.8–42% in patients with lumbar spinal stenosis (3). It has been recognized that RNRs develop as a response to lumbar spinal stenosis (4). The purpose of the study was to investigate clinical efficacy of posterior lumbar interbody fusion and internal fixation of patients with lumbar spinal stenosis and RNRs. Also, at the first time, we use pattern diagrams and their corresponding MRI images to discuss a possible classification of RNRs which includes Upper, Lower, and Intermediate RNRs (Figures 1–3).

## Methods

### General Information

Between January 2009 to December 2014, 30 patients with lumbar spinal stenosis with evidence of RNRs on MRI were treated in our hospital with posterior lumbar decompression and instrumented interbody fusion. Eleven men and 19 women (mean age, 57.6 years) with lumbar spinal stenosis and RNRs were included in this study. The ethics committee of Ningbo No.6 Hospital approved this study. The inclusion criteria included: (1) cauda equina redundancy; (2) intermittent neurological symptoms (3) history of neurogenic claudication; (4) MRI or CT confirmed central spinal canal stenosis (central sagittal diameter <10 mm, or dural sac area <100 mm^2^) (5, 6) (5) non-surgical treatment was ineffective. Exclusion criteria included: (1) spinal arteriovenous malformation, arteriovenous malformation; (2) intraspinal tumor; (3) death or loss of follow-up due to non-spinal diseases. There were 11 males and 19 females with an average age of 57.6 years (range, 49–70 years). The presurgical course of disease ranged from 2 months to 5 years, with an average of 15.1 months. There were 8 patients with lumbar spondylolisthesis, 6 with single level stenosis, 14 with two level stenosis, 8 with three level stenosis, and 2 with four level stenosis. The cauda equina was located above the stenosis in 22 cases, below it in 5 cases, and within it in 3 cases.

### Surgical Methods

After general anesthesia was induced, patients were placed into the prone position. The skin was cut longitudinally centering over the spinous processes of the lumbar spine. The fascia was divided on either side of the spinous processes, exposing the lamina, facet joints, and transverse processes. Pedicle screws were placed using anatomic landmarks and screw tracts were tapped prior to placement of appropriate length pedicle screws (Kangsheng, China). Complete laminectomy with lateral recess and foraminal decompression was performed to expand the volume of the spinal canal and neural foramen as much as possible. Thickened and calcified ligamentum flavum and facet joints hypertrophy was addressed during the decompression and compressive portions of the intervertebral disc were removed to completely decompress redundant nerve roots and cauda equina. The upper and lower endplates at each fusion level were cleaned of residual nucleus pulposus with a curette and cartilaginous endplate removed with a shaver. The intervertebral space was washed with isotonic saline before it was packed with autologous local bone graft and an interbody fusion cage filled with bone graft (Grafton, Medtronic, United States). Precut, pre-contoured rods were placed (Kangsheng, China). A drain was placed before the wound was closed in layers using absorbable suture.

### Post-operative Treatment

Perioperative treatment included protocols to prevent infection with routine antibiotics, low-dose steroid administration, and prophylactic protection of gastric mucosa. After patients had recovered from anesthesia, the patients were encouraged to actively move their lower extremity with ankle joint flexion and extension exercises and straight leg raises. The drainage tube was removed according to the drainage volume (24 h flow rate <50 m1). After 3–5 days, patients wore a lumbar brace to ambulate. Lumbar MRI was obtained 1 week after surgery. Patients began exercises to strengthen paraspinal and abdominal muscles 6 weeks after the operation.

### Evaluation Standard

The operative time, intraoperative blood loss, post-operative complications, visual analog scale (VAS), Oswestry Disability Index (ODI), and Japanese Orthopedic Association (JOA) score were compared before and after surgery. The first two post-operative improvement rates were calculated for most measures as: [(pre-treatment score – post-treatment score) / pre-treatment score] × 100%. JOA score post-operative improvement rate was calculated as: (post-treatment score – pre-treatment score) / (29 – before treatment) × 100%. Overall efficacy evaluation criteria: Fischgrund criteria was used to determine the surgical efficacy: excellent: waist or leg pain symptoms completely or almost completely disappeared, daily activities no longer affected; good: post-operative symptoms improved significantly, occasional low back pain and leg numbness, daily activities were not affected; fine: post-operative symptoms improved, intermittent episodes of low back pain or numbness of lower limbs, daily activities affected; poor: post-operative symptoms did not improve or improved and then returned to pre-operative level, daily activities were significantly affected.

### Statistical Analysis

The SPSS software version 20.0 (IBM Corporation, NY, USA) was used in the statistical analysis, and the paired sample *t*-test was used. A significance level of *P* < 0.05 was used.

## Results

### Clinical Efficacy

**Figure 4:**
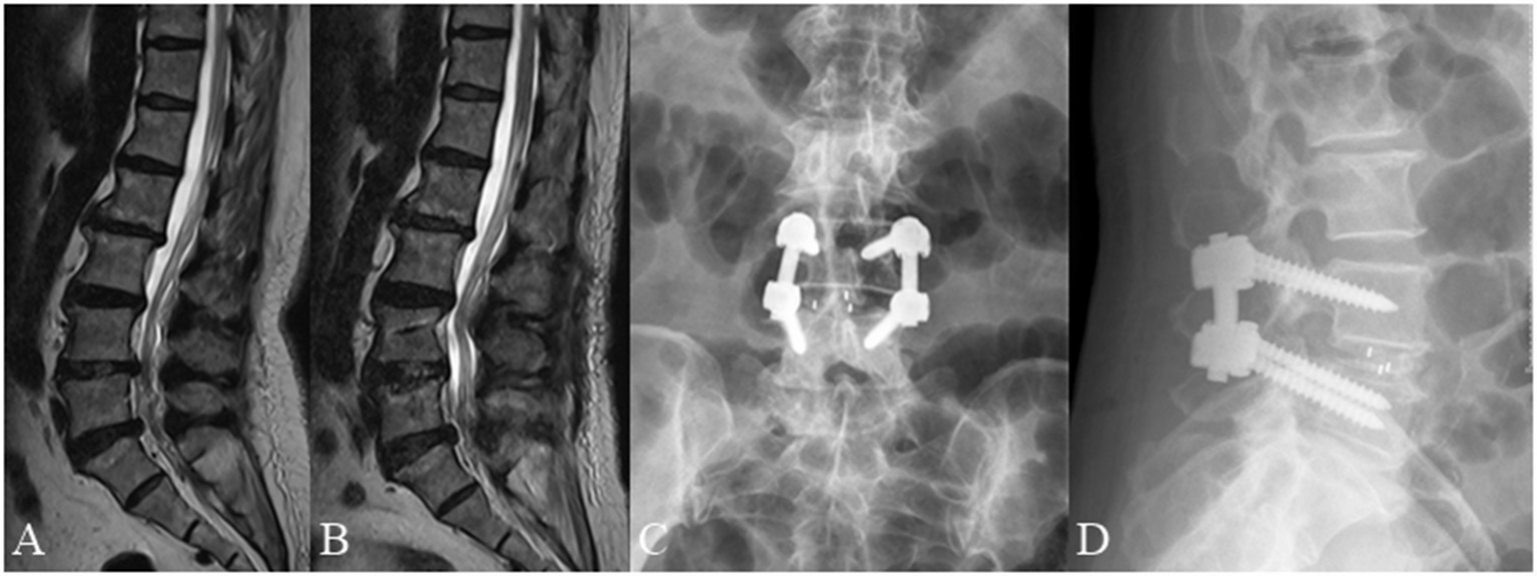
A 66 year old female complained of low back pain for 3 months underwent posterior lumbar decompression and instrumented interbody fusion at L4/5. **(A)** Pre-operative lumbar MRI showed that the nerve roots were redundant above the L4/5 intervertebral disc. **(B)** Post-operative lumbar MRI obtained 3 days after surgery demonstrated significant relief of RNRs. **(C,D)** Lumbar radiographs obtained 3 months after surgery demonstrate internal fixation in good position without cage subsidence or migration.

**Figure 5:**
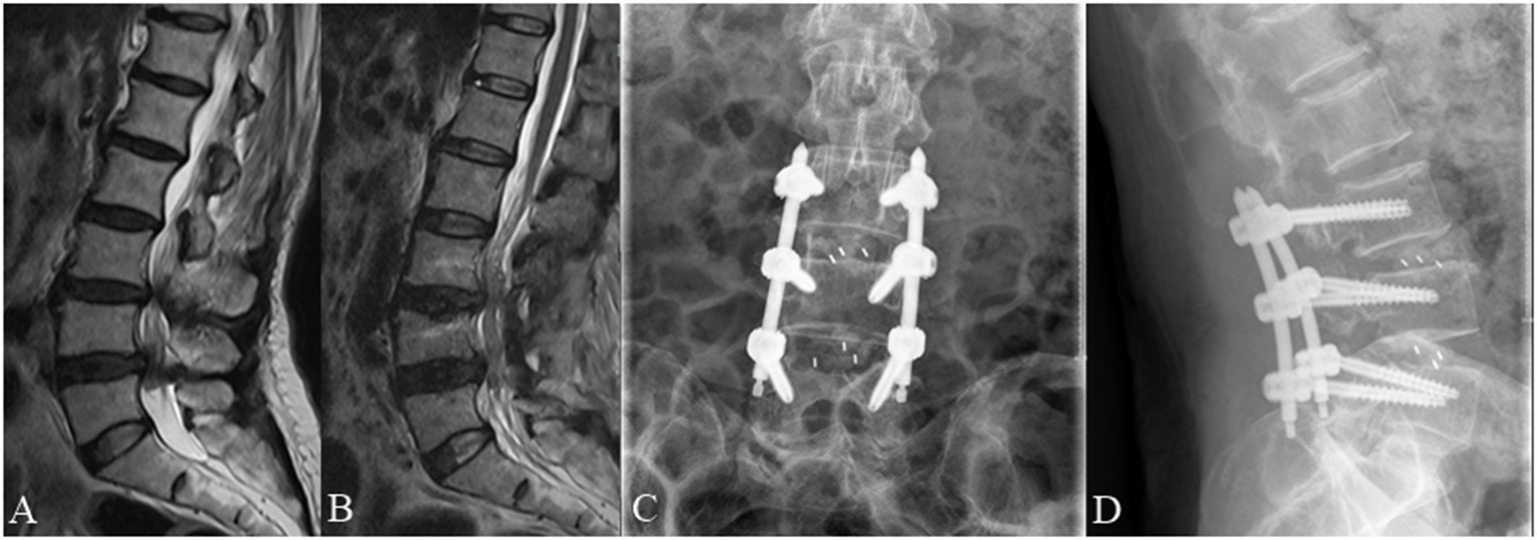
A 70 year old female complained of low back pain and right lower extremity pain for 2 months before she underwent posterior lumbar interbody fusion at L3/4 and L4/L5. **(A)** Pre-operative lumbar MRI showed that the nerve roots were redundant between the L3/4 and L4/L5 intervertebral disc. **(B)** Post-operative MRI obtained 3 days after surgery showed significant improvement with resolution of RNRs. **(C,D)** Lumbar radiographs obtained 3 months after surgery demonstrate internal fixation in good position without cage subsidence or migration.

**Figure 6:**
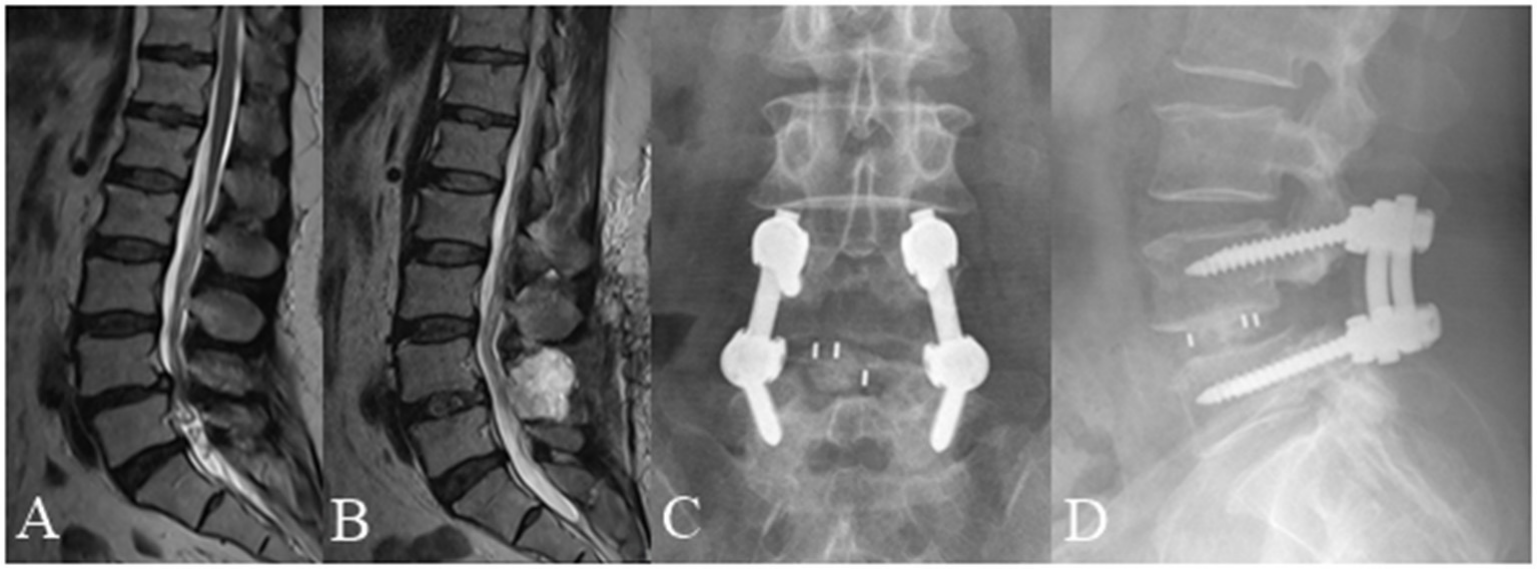
A 59 year old female complained of low back pain and left and right lower extremity pain for 3 months underwent posterior lumbar interbody fusion at L4/5. **(A)** Pre-operative lumbar MRI showed that the nerve roots were redundant below the L4/5 intervertebral disc. **(B)** Post-operative MRI obtained 3 days after surgery showed significant improvement with resolution of RNRs. **(C,D)** Lumbar radiographs obtained 3 months after surgery demonstrate internal fixation in good position without cage subsidence or migration.

All 30 patients were followed for between 12 and 30 months with an average follow up of 23.3 months (Tables 1, 2). The operative time was between 85 and 220 min with an average time of 130 min. Intraoperative estimated blood loss (EBL) ranged from 300 to 1,000 ml with an average EBL of 545 ml. Radiologists confirmed RNRs of the cauda equina were present in all 30 patients. Redundant nerve roots were located above the level of the stenosis (Upper RNRs) in 21 cases (70%), below it (Lower RNRs) in 6 cases (20%), and between the stenosis levels (Intermediate RNRs) in 3 cases (10%). Redundant nerve roots disappeared in all cases on post-surgical MRI (Figures 4–6). Pre-operative low back pain VAS, leg pain VAS, ODI, and JOA score were 6.4 ± 0.9, 7.1 ± 0.8, 43.0 ± 2.2, and 10.3 ± 2.6, respectively. Low back pain VAS, leg pain VAS, ODI, and JOA score at 3 months after surgery were (1.4 ± 0.5), (1.6 ± 0.6), (13.0 ± 1.6), and (25.0 ± 1.8), respectively. These differences for all three variables were statistically significant (*P* < 0.05). The improvement rate of VAS for low back pain was 77.1 ± 9.0%; the improvement rate of VAS for leg pain was 77.6 ± 9.1%; the improvement rate for ODI score was 69.8 ± 3.6%; the improvement rate for JOA score was 78.3 ± 9.1%. At 12 months after surgery, the interbody fusion rate was 83.3%. Efficacy evaluation demonstrated excellent outcome in 7 cases, good outcome in 13 cases, fair outcome in 8 cases, and symptoms were unchanged in 2 cases. The good or excellent rate was 66.7%. After 3 months, the patients were allowed an unrestricted activity level.

**Table 1:** Comparison of pre-operative and 3 month post-operative back pain VAS, leg pain VAS, ODI, and JOA scores (average ± standard deviation).

**Time**	**Back pain VAS**	**Leg pain VAS**	**ODI score**	**JOA score**
Pre-operative	6.4 ± 0.9	7.1 ± 0.8	43.0 ± 2.2	10.3 ± 2.6
Post-operative	1.4 ± 0.5	1.6 ± 0.6	13.0 ± 1.6	25.0 ± 1.8
*t*-value	27.22	30.07	70.26	27.97
*P*-value	<0.05	<0.05	<0.05	<0.05

*VAS, visual analog scale; ODI, Oswestry Disability Index; JOA, Japanese Orthopedic Association Scores*.

**Table 2:** Patient characteristics.

**Variable**	***n* = 30**
**Sex (** ***n*** **, %)**	
Male	18 (60%)
Female	12 (40%)
Age (x ± s)	61.47 ± 8.00
**Stenosis level (** ***n*** **, %)**	
L1/2	2 (6.7%)
L2/3	3 (10%)
L3/4	12 (40%)
L3–L5	2 (6.7%)
L4/5	10 (33.3%)
L4-S1	1 (3.3%)
**Location of RNRs (** ***n*** **, %)**	
Lower	6 (20%)
Intermediate	3 (10%)
Upper	21 (70%)
Blood loss (ml) (median, interquartile range)	500 (400–625)
**Outcome (** ***n*** **, %)**	
Unchanged	2 (6.7%)
Fair	8 (26.7%)
Good	13 (43.3%)
Excellent	7 (23.3%)

### Post-operative Complications

The surgical incisions of all patients healed uneventfully. No nerve root injury, cauda equina injury, infection, cerebrospinal fluid leakage, lower extremity venous thrombosis, subsidence, or displacement of intervertebral cage were documented.

## Discussion

**Figure 7:**
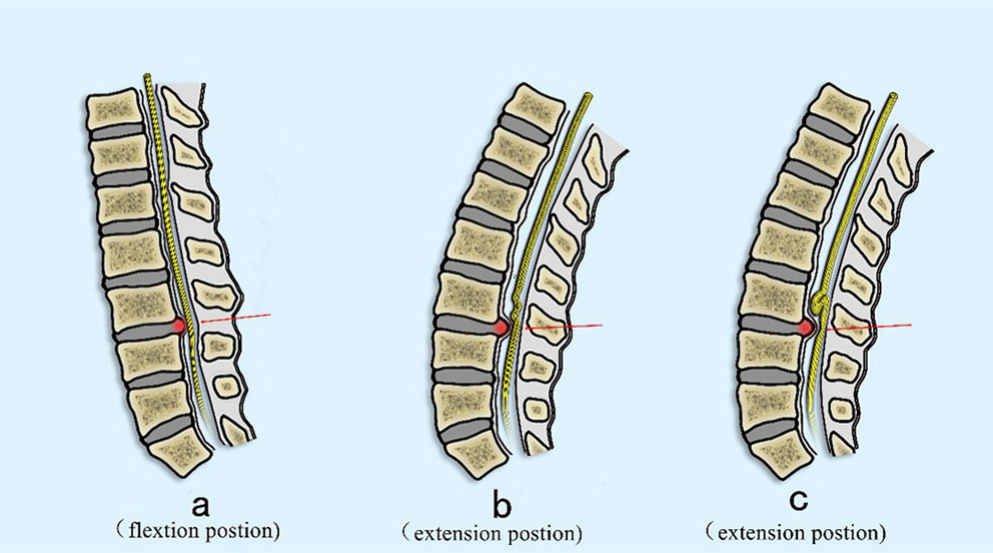
Proposed mechanism of the development of RNRs. **(A)** The nerve is pulled proximally by the flexed position commonly seen in patients with symptomatic spinal stenosis (arrow). **(B)** The nerve becomes relaxed in the extended position, but still is deformed at the site of maximal stenosis secondary to a disc extrusion (red arrow). **(C)** In an extension position, the nerve is redundant cranial to the compressed section but cannot redistribute past the compressed section due to stenosis (red arrow).

Redundant nerve roots are a common finding in which elongated, tortuous, or serpiginous nerve roots are present in the subarachnoid space (7). This condition has a clear causal relationship with central spinal stenosis, which is of great importance for the diagnosis of lumbar spinal stenosis (8). Studies (4) have shown that the occurrence rate of RNRs in patients with lumbar spinal stenosis is between 33.8 and 42.3%, with risk factors including advanced age, female gender, and patients with severe neurological symptoms (9–11). Clinical manifestations are mostly similar to those seen with spinal stenosis such as leg pain with intermittent claudication and low back pain. Besides, the pathogenesis of RNRs is still unclear. Suzuki et al. (3) proposed a possible mechanism for the formation of RNRs in the 1980s: RNRs are most likely the pathological result of a chronic compression force at the level of spinal canal stenosis (12). Their study with histopathologic evaluation identified nerve fiber degeneration and neuronal loss due to continuous mechanical compression of the nerve roots, which were confined to the nerves in the narrowed section of the spinal canal [(13, 14); Figure 7]. In the present study, 30 patients underwent routine imaging with lumbar MRI after lumbar interbody fusion surgery. The lumbar spine MRI was obtained and reviewed 1 week after surgery with disappearance of RNRs noted in all cases. The rate of excellent and good outcomes was 66.7% based on evaluation using Fischgrund criteria.

Kirkaldy-Willis and Hill (15) noted that all treatments for spinal degenerative diseases (including surgical treatment) can alleviate the clinical symptoms but fail to deal with underlying fundamental degenerative processes to cure the disease. In this light, Spengler (16) stated that indications for surgery depend on the patient's need for improved quality of life in the future. Therefore, patients with RNRs on MRI are indicated for surgery, which doesn't rely on the presence of RNRs but is based on the presence of: severe pain in the lower extremities causing difficulty with activities of daily living, signs of objective nerve damage such as weakness, muscle atrophy, and neurogenic claudication symptoms which limit walking or standing of patients for longer than 3 months and can't be alleviated via non-surgical treatment.

The purpose of surgical treatment for patients with lumbar spinal stenosis is to enlarge the volume of the spinal canal, relieve nerve compression, and reconstruct the stability of the spine (17). For the group of patients who predominantly suffer from claudicatory leg pain, the key element of the surgery is to successfully decompress the spinal nerves, thus encouraging us to treat these patients with posterior lumbar instrumented interbody fusion with total laminectomy. The advantage of this surgery is that extensive spinal canal decompression can be achieved by removing the lamina, facet joints, spinous processes, and associated ligaments.

Patients with RNRs often have adhesions around the cauda equina, thus requiring careful and meticulous technique when performing decompression of redundant nerve roots. Besides, it should be mentioned that thorough decompression is required to remove all elements of the spinal canal which can cause compression of nerve roots (bone spurs, hypertrophic ligamentum flavum, displaced intervertebral disc material, and hypertrophic facet joints) to restore nerve root freedom. For patients with lumbar spine MRI showing foraminal stenosis, decompression of the nerve root canal is also needed.

Due to the presence of RNRs, attention has been paid not only to abnormalities of the bone and soft tissues that make up the spinal canal, but also to the condition of the nerve roots within the dural sac. In patients with RNRs, the nerve root may be more tightly compressed. In the flexion position, the nerve root is elongated, and it is easy to produce distortion, entanglement, and adhesions over time. Furthermore, with the demyelination and fibrosis which may be associated with severe stenosis, the symptoms of low back and leg pain may be more severe and the prognosis after surgery will be worse. Ono et al. (9) pointed out that patients with RNRs are also featured with worse clinical manifestations and symptoms compared with other lumbar spinal stenosis patients. Chen et al. (18) showed that in 93 patients with lumbar spinal stenosis who underwent surgery, RNR patients had a poorer prognosis in post-operative pain, limb numbness, and walking ability than those without RNRs.

The present study showed that RNRs of the cauda equina are not uncommon in patients with lumbar spinal canal stenosis. RNRs of the cauda equine are frequently observed in the superior of the stenosis level (Upper) but can also be observed in both inferior and superior (Intermediate), and less frequently in inferior localizations (Lower) only (19). So, it is of great importance to classify different types of RNRs. To make a clear and vivid presentation, we use pattern diagrams and their corresponding MRI images to discuss a possible classification of RNRs which includes Upper, Lower and Intermediate RNRs (Figures 1–3).

This study has several limitations. Due to the small sample size of this case group, it is difficult to comment on its generalizability. In addition, because of the lack of a control group, there may be bias in the evaluation of our surgical treatment, and our results can't be compared against other surgical or non-surgical options. In the future, the studies on the relationship of nerve root redundancy and the lumbar spinal stenosis degree with outcome after different surgical procedures should be conducted.

## Conclusion

Posterior lumbar decompression and instrumented interbody fusion and internal fixation can effectively decompress stenotic segments and free nerve roots, alleviate the redundancy of the nerve roots and improve low back and leg pain. Moreover, while RNRs are relatively common in patients with lumbar spinal stenosis, patients with RNRs often present with worse clinical symptoms and prognosis (20, 21). Therefore, when evaluating patients with lumbar spinal stenosis, the lumbar spine MRI should be used routinely to not only assess the degree and location of canal stenosis but also to evaluate the position and state of the nerve roots in order to provide a clearer understanding and better prognostic guidance when considering surgical options.

## Data Availability Statement

The original contributions presented in the study are included in the article/supplementary material, further inquiries can be directed to the corresponding author/s.

## Ethics Statement

The studies involving human participants were reviewed and approved by Ningbo No.6 Hospital. Written informed consent for participation was not required for this study in accordance with the national legislation and the institutional requirements. Written informed consent was obtained from the individual(s) for the publication of any potentially identifiable images or data included in this article.

## Author Contributions

All authors listed have made a substantial, direct and intellectual contribution to the work, and approved it for publication.

## Conflict of Interest

The authors declare that the research was conducted in the absence of any commercial or financial relationships that could be construed as a potential conflict of interest.

## Publisher's Note

All claims expressed in this article are solely those of the authors and do not necessarily represent those of their affiliated organizations, or those of the publisher, the editors and the reviewers. Any product that may be evaluated in this article, or claim that may be made by its manufacturer, is not guaranteed or endorsed by the publisher.
